# Complex Multimeric [FeFe] Hydrogenases: Biochemistry, Physiology and New Opportunities for the Hydrogen Economy

**DOI:** 10.3389/fmicb.2018.02911

**Published:** 2018-12-04

**Authors:** Kai Schuchmann, Nilanjan Pal Chowdhury, Volker Müller

**Affiliations:** Molecular Microbiology and Bioenergetics, Institute of Molecular Biosciences, Johann Wolfgang Goethe University, Frankfurt am Main, Germany

**Keywords:** hydrogenase, formate dehydrogenase, CO_2_ reduction, electron bifurcation, hydrogen production, acetogenesis, clostridia, hydrogen storage

## Abstract

Hydrogenases are key enzymes of the energy metabolism of many microorganisms. Especially in anoxic habitats where molecular hydrogen (H_2_) is an important intermediate, these enzymes are used to expel excess reducing power by reducing protons or they are used for the oxidation of H_2_ as energy and electron source. Despite the fact that hydrogenases catalyze the simplest chemical reaction of reducing two protons with two electrons it turned out that they are often parts of multimeric enzyme complexes catalyzing complex chemical reactions with a multitude of functions in the metabolism. Recent findings revealed multimeric hydrogenases with so far unknown functions particularly in bacteria from the class Clostridia. The discovery of [FeFe] hydrogenases coupled to electron bifurcating subunits solved the enigma of how the otherwise highly endergonic reduction of the electron carrier ferredoxin can be carried out and how H_2_ production from NADH is possible. Complexes of [FeFe] hydrogenases with formate dehydrogenases revealed a novel enzymatic coupling of the two electron carriers H_2_ and formate. These novel hydrogenase enzyme complex could also contribute to biotechnological H_2_ production and H_2_ storage, both processes essential for an envisaged economy based on H_2_ as energy carrier.

## Introduction

Molecular hydrogen (H_2_) is only present in trace concentrations (550 parts per billion) in the Earth’s lower atmosphere (Novelli et al., 1999). Nevertheless, it plays an essential part in the biogeochemical cycles of other elements such as carbon and is a major constituent of the microbial metabolism. For example, H_2_ is an important electron donor for methane formation in anoxic environments (Schink, 1997; Thauer et al., 2008). Here, too, steady state concentrations are very low (pH_2_ < 10 Pa) but the turnover of H_2_ is very high (150 million tons of H_2_ of biological origin are estimated to be produced in anoxic ecosystem annually to fuel methanogenesis) (Thauer et al., 2008, 2010). In anoxic ecosystems, the major role of H_2_ is electron transfer between the different participants of the food chain, e.g., transfer of electrons generated by primary fermenters to methanogens (Stams and Plugge, 2009; Schink et al., 2017). To produce or consume H_2_ nature has evolved complex metalloenzymes, hydrogenases, which catalyze one of the simplest chemical reaction, reversible oxidation of H_2_ into two protons and two electrons:

(1) $$H_{2}⇌2H^{+}+2e^{-}$$

Hydrogenases are widespread in nature and can be found in all domains of life. Based on their phylogeny, they can be classified into three distinct classes that are named by the metal ions contained in their active sites as [NiFe]-, [FeFe]-, and [Fe] hydrogenases (Vignais et al., 2001; Vignais and Colbeau, 2004). [NiFe] hydrogenases have been found in bacteria and archaea, [FeFe] hydrogenases in bacteria and some eukaryotes, and [Fe] hydrogenases only in archaea (Vignais and Billoud, 2007). Even though [NiFe]- and [FeFe] hydrogenases have evolved independently, the complex metal centers responsible for catalysis share many features. The metal ions are ligated by inorganic CO and CN^-^ ligands and are bridged by sulfur atoms. H_2_ can reach the active sites that are buried within the enzymes by a hydrophobic gas channel and the electrons that result from H_2_ oxidation are in both enzyme classes transferred to an [4Fe–4S] cluster adjacent to the metal center (Happe et al., 1997; Pierik et al., 1998; Fontecilla-Camps and Ragsdale, 1999). The third class of hydrogenases, the [Fe] hydrogenases which are only found in methanogenic archaea differ not only by their architecture of the active site, but also by the catalyzed reaction that does not result in the release of electrons to an iron–sulfur cluster, but the direct transfer to the cosubstrate methenyltetrahydromethanopterin (Shima and Thauer, 2007; Shima et al., 2008). In this review, we will mainly focus on hydrogenases of the [FeFe] class. Enzymes from this class are widespread in anaerobic prokaryotes and play important roles in the energy and carbon metabolism in anoxic ecosystems. Crystal structures have been reported for three enzymes, namely from *Clostridium pasteurianum* (Peters et al., 1998), *Desulfovibrio desulfuricans* (Nicolet et al., 1999) and the eukaryotic algae *Chlamydomonas reinhardtii* (Mulder et al., 2010) and show high similarity in the overall structure and the architecture of the active site. Details on the crystal structures and the reaction mechanism have been described in excellent and comprehensive reviews elsewhere and will therefore not been repeated here except for the architecture of the cofactors in the *C. pasteurianum* enzyme that we will discuss in the context of electron bifurcation later (Vignais and Colbeau, 2004; Thauer et al., 2010; Lubitz et al., 2014). Interestingly, despite the apparent high similarity of the “core” hydrogenase subunit responsible for H_2_ oxidation and production, [FeFe] hydrogenases show a remarkable diversity with respect to the auxiliary subunits that can be found in most enzymes and an even higher diversity can be predicted from genome sequence data. The auxiliary subunits follow a very modular structure and add multiple functions to the core subunit. These functions can include electron transfer to soluble electron carriers, coupling of H_2_ oxidation/production to other chemical reactions, coupling to energy conservation by coupling the electron transfer to the generation of a transmembrane ion potential, or utilization of the novel energetic coupling mechanism of flavin-based electron bifurcation (FBEB) to overcome energetic limitations of the electron transfer (Buckel and Thauer, 2018a,b; Müller et al., 2018). A notable diversity of multimeric [FeFe] hydrogenases can be found especially in the strictly anaerobic Gram-positive bacteria of the class Clostridia within the phylum Firmicutes (Calusinska et al., 2010; Schmidt et al., 2010). Recent discoveries have revealed multimeric hydrogenases with remarkable and so far undescribed functions and catalytic properties, solving important questions of the metabolism and ecosystem functioning in anoxic environment. In addition, recently described multimeric hydrogenases provide interesting opportunities for biotechnological applications. H_2_ is a candidate energy carrier that could replace fossil fuels for storage and transportation of energy generated from renewable sources such as wind or solar power (Dunn, 2002; Brandon and Kurban, 2017). However, for an economically viable H_2_ economy many obstacles need to be overcome such as efficient methods for H_2_ production and technologies for storage and transportation of the volatile and explosive gas (Service, 2004; Preuster et al., 2017a).

Here, we review the recent findings of novel complex multimeric hydrogenases and especially their function in the microbial H_2_ metabolism. These include enzymes using the novel energy coupling mechanism of FBEB enabling otherwise endergonic H_2_ production from NADH or the otherwise endergonic electron transfer from H_2_ to the iron–sulfur protein ferredoxin. The second part will focus on the recently discovered formate dehydrogenase coupled hydrogenases enabling direct CO_2_ reduction with H_2_ or H_2_ evolution from formate. In addition, both hydrogenase types will be discussed in the context of their biotechnological potential for the H_2_ economy.

## H_2_ Functioning as Electron Carrier in Anoxic Ecosystems

H_2_ can be utilized by many organisms as electron donor. Recently, it has been reported that microorganisms are even capable of utilizing the low atmospheric concentrations of H_2_ (Greening et al., 2014a,b). Nevertheless, the most prominent functions of H_2_ are found in anoxic ecosystems where it is rapidly produced and consumed by microorganisms resulting in a large turnover. H_2_ connects different parts of the food web to allow full remineralization of organic material. When complex polymeric organic material (polysaccharides, proteins, nucleic acids, lipids) enter anoxic ecosystems it is typically first hydrolyzed by exoenzymes followed by the partial oxidation by primary fermenting microorganisms into different fermentation products such as lactate, alcohols, short chain fatty acids, acetate, formate, CO_2_ and H_2_. In the absence of suitable external electron acceptors, the latter compounds (acetate, formate, CO_2_ and H_2_) can be directly converted by methanogenic archaea into methane (Schink, 1997; Thauer et al., 2008). Further oxidation of the other compounds leads to an energetic problem that can only be solved by the concerted cooperation of secondary fermenting organisms with methanogens in a process call syntrophy (Schink, 2002; Sieber et al., 2012; Morris et al., 2013). Secondary fermenters oxidize their substrates typically to acetate as end product coupled to oxidation of protons to H_2_ to reoxidize their electron carriers. However, under standard conditions these reactions are endergonic and do not provide energy for the cell. During substrate oxidation, electrons are typically transferred to either ferredoxin, a small iron–sulfur cluster containing protein with a very negative redox potential (E°’ = -400 to -500 mV), or NAD^+^ with a more positive redox potential (E°’[NAD^+^/NADH] = -320 mV) (Thauer et al., 1977). Proton reduction can be used to recycle these electron carriers. The redox potential of the H^+^/H_2_ couple is, under standard conditions, -414 mV. Therefore, while H_2_ production from reduced ferredoxin is an exergonic reaction, H_2_ formation from NADH represents a strong energetic barrier for the cells since it is a highly endergonic reaction (Thauer et al., 1977). Two possible mechanisms have evolved to overcome this problem and allow H_2_ formation from NADH. In the classical view, H_2_ oxidizing methanogens lower the H_2_ partial pressure to very low values (1–10 Pa H_2_) resulting in a more positive redox potential of the H^+^/H_2_ couple (above -300 mV) thus rendering H_2_ formation from NADH an exergonic reaction (Schink, 2002; Sieber et al., 2012; Morris et al., 2013). Recently, another mechanism has been discovered that solves the problem of NADH reoxidation with protons as acceptor within one enzyme. Complex multimeric hydrogenases are necessary to catalyze this reaction that solves the energetic problem by energetically coupling the endergonic electron transfer to a second exergonic redox reaction, a process called FBEB. As we will see later, these enzymes not only participate in the metabolism of secondary fermenting organisms but can also be found in primary fermenters, thereby increasing the energy yield that can be conserved from a given substrate, or, in the reverse, be found in acetogenic bacteria to allow ferredoxin reduction with H_2_ as electron donor.

## The Concept of Flavin-Based Electron Bifurcation

In 2008 a new energy coupling mechanism called FBEB was first discovered in an enzyme complex of an electron-transferring flavoprotein and a butyryl-CoA dehydrogenase (Etf/Bcd) (Herrmann et al., 2008; Li et al., 2008). In FBEB, an electron pair from an electron donor such as NADH is split toward two different one-electron acceptors, one with a more positive redox potential and another with a much lower redox potential than that of the electron donor. It was proposed and later proven that electron transfer to a positive redox potential (exergonic reaction) sustains the movement of an electron to a more negative redox potential (endergonic reaction). In case of Etf/Bcd, the positive electron redox potential acceptor was crotonyl-CoA (E^0^’ = -10 mV) and the negative redox potential acceptor was ferredoxin (E^0^’ = -420 mV). NADH (E^0^’ = -320 mV) is the electron donor. The complete reaction catalyzed by the protein complex is

(2) $$\mathrm{Crotonyl}-\mathrm{CoA}+2\mathrm{NADH}+{\mathrm{Fd}}_{\mathrm{ox}}\to \mathrm{Butyryl}-\mathrm{CoA}+2{\mathrm{NAD}}^{+}+{\mathrm{Fd}}_{\mathrm{red}}$$

The enzyme complex contained FAD as the only cofactor that was essential for the activity (Chowdhury et al., 2014; Demmer et al., 2017). Hence, the name originated as FBEB. FBEB was drawn in analogy to the quinone-based electron bifurcation (QBEB) of the cytochrome bc_1_ complex of the respiratory chain, which was discovered 43 years ago by Peter Mitchell, in which the oxidation of reduced ubiquinone (UQH_2_) by the high potential cytochrome c_1_ by one electron allows the reduction of low-potential cytochrome b_L_ and further UQ inside the membrane (Mitchell, 1975). The process is repeated twice that allows four protons to be released outside of the cell and doubling the amount of energy conserved and electrons finally flow down to oxygen (terminal electron acceptor) to reduce oxygen to water.

## H_2_ Production From NADH: Electron-Bifurcating Hydrogenases for H_2_ Evolution

Soon after the discovery of FBEB in Clostridia, the first hydrogenase was reported that utilizes the mechanism of FBEB, however, in the physiological context in the reverse direction (named electron confurcation) (Schut and Adams, 2009). The enzyme was discovered in the hyperthermophilic and anaerobic bacterium *Thermotoga maritima.* The bacterium ferments one mole of glucose by the classical Embden–Meyerhof–Parnas pathway to two moles of CO_2_, two acetate and four moles of H_2_ (Schroder et al., 1994). During its metabolism, both NADH and reduced ferredoxin are generated, however, for decades the link to the oxidation of these electron carriers to H_2_ production remained obscure (Wrba et al., 1990; Blamey and Adams, 1993). The trimeric [FeFe] hydrogenase was isolated and could be assayed by coupling reduction of viologen dyes with H_2_. However, the enzyme did not use either reduced ferredoxin or NADH as sole electron donor (Verhagen et al., 1999). Though reduced clostridial ferredoxin (E^0^’ = -420 mV) alone can reduce protons to H_2_ (E^0^’ = -414 mV), NADH cannot. It was an enigma since the discovery of fermentative H_2_ production to how H_2_ is produced from NADH. The solution was FBEB: exergonic electron flow from reduced ferredoxin to H^+^ that drives endergonic electron flow from NADH to H^+^, according to:

(3) $$\mathrm{NADH}+{\mathrm{Fd}}_{\mathrm{red}}+3H^{+}⇌{\mathrm{NAD}}^{+}+{\mathrm{Fd}}_{\mathrm{ox}}+2H_{2}$$

**FIGURE 1:**
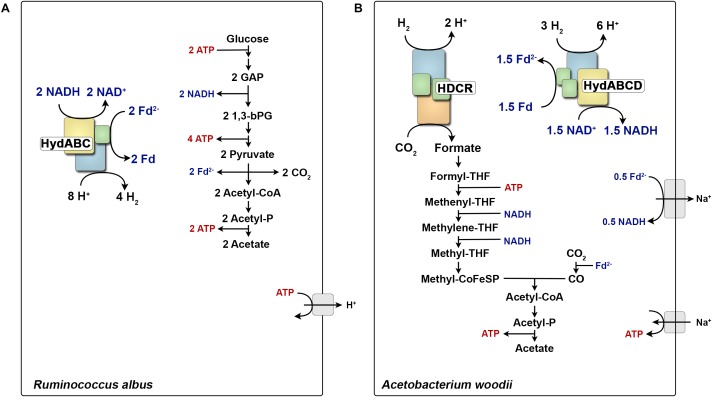
Role of multimeric hydrogenases in the energy metabolism of *Ruminococcus albus* **(A)** and *Acetobacterium woodii* **(B)**. During glucose fermentation, *R. albus* oxidizes glucose to two molecules acetate. All generated reducing equivalents are reoxidized by the electron confurcating hydrogenase HydABC. In contrast, *A. woodii* can grow with H_2_ + CO_2_ as substrates and forms acetate as major end product. HDCR catalyzes the first step of the WLP from CO_2_ to formate. All reducing equivalents are provided from H_2_ oxidation catalyzed by the electron bifurcating hydrogenase HydABCD. Fd^2-^, reduced ferredoxin; CoA, coenzyme-A; THF, tetrahydrofolate; CoFeSP, corrinoid–iron–sulfur protein; GAP, glyceraldehyde 3-phosphate; 1,3-bPG, 1,3-bisphosphoglycerate.

The hydrogenase of *T. maritima* is now the classic example where reduced ferredoxin drives H_2_ evolution from NADH. The enzyme oxidizes NADH and ferredoxin simultaneously in a 1:1 ratio to produce H_2_. In this case both electrons from NADH and reduced ferredoxin are converged to reduce protons to H_2_. This mode of electron converging from different sources of electron donor (NADH and Fd_red_) to a single electron acceptor (protons) is now called electron confurcation. A similar lifestyle and metabolic pathway is also observed in the rumen bacterium *Ruminococcus albus* (Zheng et al., 2014). Similar to *T. maritima*, when grown in continuous culture the bacterium produces the same amounts of H_2_ and acetate from glucose (Figure 1A). Also, the acetogenic model bacterium *Acetobacterium woodii* is suggested to use a confurcating hydrogenase to evolve H_2_ from organic substrates (Schuchmann and Müller, 2012; Bertsch et al., 2015; Kremp et al., 2018). This mode of H_2_ evolution by electron confurcating hydrogenases is important for energy conservation in these fermenting bacteria. ATP is synthesized by substrate level phosphorylation at the phosphoglycerate kinase and pyruvate kinase reactions and NADH is formed by NAD-specific glycerinaldehyde-3-phosphate-dehydrogenase. The redox pool could for example be balanced by reducing pyruvate to lactate, where 2 NADH will be consumed. However, this pathway will produce 2 ATP less. Rather the bacteria maintain its redox balance by producing two moles of acetyl-CoA *via* the pyruvate-ferredoxin oxidoreductase and finally releases 2 ATP catalyzed by the acetate kinase. This mode of metabolism leaves 2 NADH and 2 reduced ferredoxin that are converted to H_2_ by FBEB hydrogenase. Hence, the presence of FBEB hydrogenases allows NADH and ferredoxin to be reoxidized with H^+^ as electron acceptor and thus increasing the ATP yield (Müller et al., 2018).

## Electron-Bifurcating Uptake Hydrogenases

In the autotrophic mode of life, organisms can synthesize most of their carbon compounds from CO_2_ using H_2_ or another electron donor as reductant. One such group are the acetogenic bacteria that are capable of producing acetate from two molecules of CO_2_ with H_2_ as the reductant *via* the reductive acetyl-CoA pathway (also known as Wood–Ljungdahl pathway, WLP) (Wood, 1991; Wood and Ljungdahl, 1991). Three model organisms that have been studied in detail are *Moorella thermoacetica, A. woodii*, and *Clostridium ljungdahlii*. As these bacteria grow simply on CO_2_ and H_2_ without any external carbon source added, this mode of metabolism must be coupled to net ATP formation.

*A. woodii* as a model organism has been studied in detail to answer how energy is conserved during acetogenesis (Schuchmann and Müller, 2014). This unearthed surprising enzyme complexes that are unique in their diversity of the reactions they catalyze as well as their working mechanisms. Three such enzyme complexes are the Rnf complex, an FBEB hydrogenase and a H_2_-dependent CO_2_ reductase. *A. woodii* employs a sodium ion-dependent ferredoxin: NAD-oxidoreductase (Rnf complex) that couples the exergonic oxidation of reduced ferredoxin (E^0^’ ∼-450 mV) with NAD^+^ (E^0^’ = -320 mV) to the generation of a transmembrane electrochemical Na^+^ gradient (Figure 1B). The difference in the redox potential of ferredoxin and NAD^+^/NADH allows the pumping of Na^+^ over the cytoplasmic membrane resulting in a Na^+^ gradient that then drives the synthesis of ATP *via* the well-characterized Na^+^ F_1_F_O_-ATP synthase (Spruth et al., 1995). Therefore, to drive energy conservation catalyzed by the Rnf complex, ferredoxin needs to be reduced. *A. woodii* solves this problem by using a FBEB uptake hydrogenase (Schuchmann and Müller, 2012) which is quite similar to the electron confurcating hydrogenases from *T. maritima* or *R. albus* (Zheng et al., 2014). However, in case of chemolitotrophic growth, H_2_ is oxidized to reduce both NAD^+^ and ferredoxin. The hydrogenase couples the exergonic oxidation of H_2_ (E^0^’ = -414 mV) with NAD^+^ (E^0^’ = -320 mV) to the endergonic reduction of ferredoxin (E^0^’ ∼-450 mV). Reduced ferredoxin is used in two different reactions, one to reduce CO_2_ to CO (E^0^’ = -520 mV) in the WLP and secondly to transfer electrons *via* the Rnf to NAD^+^, resulting in a Na^+^ gradient (Biegel and Müller, 2010, 2011; Biegel et al., 2011). However, reaching the very low reduction potential for CO_2_ to CO reduction is difficult even at very high H_2_ partial pressure (10^5^ Pa). FBEB provides an elegant solution by providing reduced ferredoxin with a more negative redox potential than the initial electron donor H_2_. The same is true when *A. woodii* is grown on methanol, where FBEB hydrogenase provides the extra reduced ferredoxin which is then used for CO_2_ reduction (Kremp et al., 2018).

The hydrogenase of *A. woodii* has been studied in the context of the autotrophic metabolism where its function is H_2_ oxidation. Besides, further insights into the non-autotrophic metabolism of *A. woodii* have revealed that the enzyme may function as a H_2_-evolving (electron bifurcating) hydrogenase as well. For example, when *A. woodii* grows on ethanol, only NADH is produced when ethanol is oxidized to acetyl-CoA. One part of the NADH is oxidized at the Rnf complex reducing ferredoxin. This reduced ferredoxin is then used with the other part of NADH by the hydrogenase to produce H_2_ for the first step of the WLP (Figure 1B) (Bertsch et al., 2015). So, the electron-bifurcating/confurcating hydrogenase from *A. woodii* provides a nice example how anaerobes have evolved their metabolic enzymes which serves the purpose of both uptake and evolving H_2_ when needed in two different modes of energy metabolism.

## Cofactor and Subunit Architecture of FBEB Hydrogenases

**FIGURE 2:**
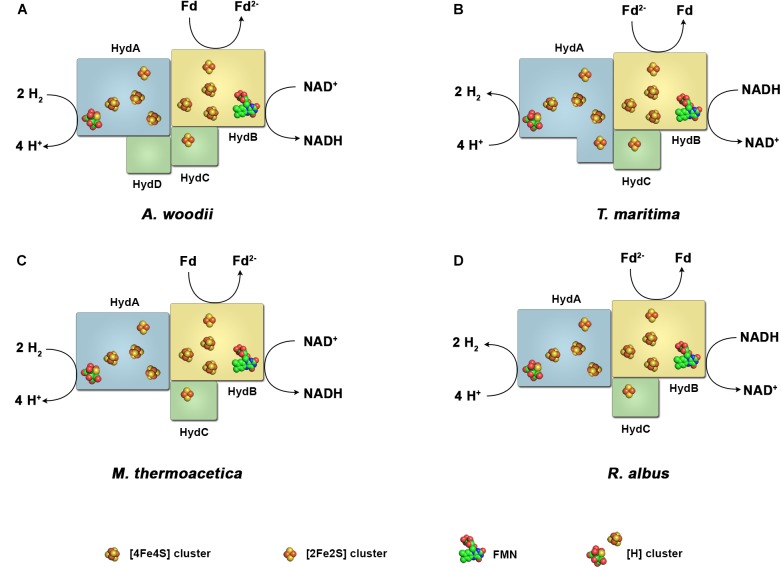
Diversity of the subunit architecture and cofactor content of electron bifurcating and confurcating hydrogenases. Enzymes of this class have been isolated and characterized from *A. woodii* **(A)**, *T. maritima* **(B)**, *M. thermoacetica* **(C)** and *R. albus* **(D)**. All enzymes contain the subunits HydA, harboring the active site, the putative flavin-containing and NAD-binding subunit HydB, and the putative electron-transferring subunit HydC. *A. woodii* contains the additional subunit HydD. The arrangement of the iron–sulfur clusters and the binding site for ferredoxin are chosen arbitrary. Fd, ferredoxin.

**FIGURE 3:**
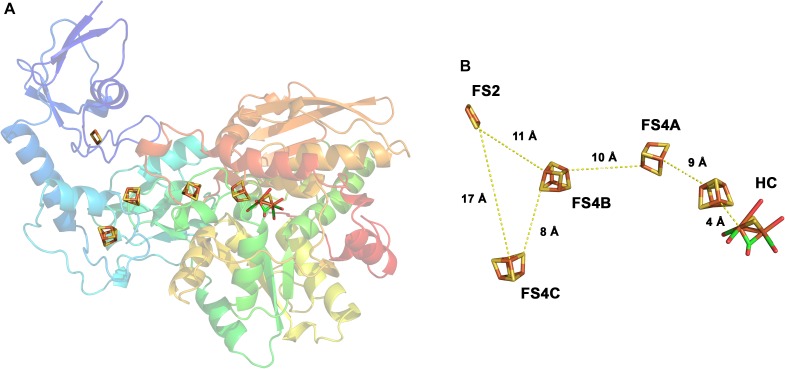
Structure of the monomeric ferredoxin-dependent hydrogenase of *C. pasteurianum*. The 3D structure has been solved at 1.8 Å resolution (1FEH, RSCB PDB database). The monomeric enzyme contains the H-cluster typical for [FeFe] hydrogenases including the auxiliary [4Fe–4S] cluster. In addition, one [2Fe–2S]- and three [4Fe–4S] clusters are bound in the enzyme **(A)**. The iron–sulfur centers are arranged in a forked architecture with cluster FS2 being too far away from FS4C for direct electron transfer **(B)**. FS2 and FS4C are both close enough to FS4B for electron transfer. HC, H-cluster. Modified from Peters et al. (1998).

The H_2_ forming hydrogenases from *T. maritima, R. albus*, and *M. thermoacetica* (Wang et al., 2013b) and the H_2_ uptake/forming hydrogenase from *A. woodii* possess quite similar subunit compositions (Figure 2). All four enzymes are composed of the three subunits Hyd A (∼64 kDa), Hyd B (∼65 kDa) and Hyd C (∼14 kDa). HydA from *T. maritima* is larger compared to its counterpart by having a size of 73 kDa putatively containing an additional [2Fe–2S] cluster. Another exception is that the *A. woodii* hydrogenase has an extra subunit HydD (∼15 kDa) which is predicted to contain no cofactor (Schuchmann and Müller, 2012, 2014). On amino acid sequence comparison, HydA finds the closest similarity to the monomeric [FeFe] hydrogenase from *C. pasteurianum* (Figure 3). The first crystal structure of the [FeFe] hydrogenase from *C. pasteurianum* CpI was reported by Peters et al. (1998). The overall structure of the core domain consists of the H-cluster (the active site of [FeFe] hydrogenases catalyzing H_2_ oxidation) including a diiron subcluster and one [4Fe–4S] cluster connected *via* a cysteine residue as found conserved in most of the [FeFe] hydrogenases. The diiron metals are coordinated by CN^-^ and CO, while the proximal Fe is linked to a cubane [4Fe–4S] cluster *via* a cysteine. The [4Fe–4S] is around 4 Å apart from the di-iron center. Apart from the H_2_-activating domain the domain interacting with the active-site domain contains two [4Fe–4S] clusters named as FS4A and FS4B (Figure 3). FS4A is 9 Å apart from the H-cluster thus the direct electron carrier to or from the H-cluster. FS4A is around 10 Å apart from FS4B cluster and thus in line to the putative electron transfer pathway. There are two additional domains, one containing a [2Fe–2S] cluster named FS2 (11 Å from FS4B), the other contains a single [4Fe–4S] cluster called FS4C. FS4C has an unusual three cysteine and one histidine ligation. The two clusters FS4C and FS2 exhibit a forked architecture, where FS2 is far away from the direct line of electron transfer from FS4C. Most probably, FS4C directly gets electron from FS4B and finally transfers electrons to ferredoxin. A recent study using protein–protein docking modeling and NMR studies of electron transfer complex formation between the photosynthetic electron-transfer ferredoxin (PetF) containing a [2Fe–2S] cluster and the hydrogenase HydA1 from the microalga *C. reinhardtii* revealed PetF to interact with HydA1 near to FS4C (Chang et al., 2007; Rumpel et al., 2015). The function of FS2 and the forked orientation of the possible electron transfer chains is puzzling, especially in the context of an electron bifurcating enzyme.

Assuming a similar structural organization and cofactor content in the FBEB hydrogenases, HydA is predicted as the catalytic subunit for H_2_ oxidation. It contains the H-cluster which is the site for H_2_ activation, one [2Fe–2S] and three [4Fe–4S] clusters. HydB has closest similarity to the NADH binding subunit NuoF of NADH-quinone oxidoreductase from *E. coli*, and is predicted to contain one FMN, one [2Fe–2S] and three [4Fe–4S] clusters. HydC, which is related to NuoE, contains only one [2Fe–2S] cluster.

## A Possible Electron Pathway in FBEB Hydrogenases

How can the electron flow from H_2_ to NAD^+^ and H_2_ to ferredoxin be energetically coupled within these multimeric hydrogenases? Until now the basis of FBEB has been revealed in other enzyme complexes like the Etf/Bcd complex from *Acidaminococcus fermentans* (Chowdhury et al., 2014, 2016), *C. difficile* (Demmer et al., 2017) and *Megasphaera elsdenii* (Chowdhury et al., 2015), LctBCD and CarCDE of *A. woodii* (Bertsch et al., 2013; Weghoff et al., 2015) and the Nfn transhydrogenase from *T. maritima* (Demmer et al., 2015), *Pyrococcus furiosus* (Lubner et al., 2017), FixABCX of *Azotobacter vinelandii* (Ledbetter et al., 2017) and HdrABC-MvhAGD from the thermophilic methanogenic archaeon *Methanothermococcus thermolithotrophicus* (Wagner et al., 2017). However, FBEB hydrogenases represent a very special case. FBEB enzymes so far all are proposed to contain a flavin having special redox properties. Flavins have three different redox potentials for the three possible redox reactions:

(4) $$FMN+1\;e^{-}⇌FMN^{\circ -}\;\;\;\;(E_{1})$$

(5) $$FMN^{\circ -}+1\;e^{-}+2\;H^{+}⇌FMNH_{2}\;\;\;\;\;(E_{2})$$

(6) $$FMN+2\;e^{-}+2\;H^{+}⇌FMNH_{2}\;\;\;\;(E_{3})$$

In “standard” flavins the midpoint potential of E_1_ is more positive than E_2_. In FBEB enzymes the flavin is supposed to have a “crossed” redox potential meaning that E_1_ is more negative than E_2_ (Nitschke and Russell, 2012). The two-electron redox potential (E_3_) is supposed to be between, meaning the average, of E_1_ and E_2_. Therefore, the flavin can be reduced in a two-electron transfer reaction by the electron donor (E_3_) followed by the first electron being transferred to the more positive electron acceptor (e.g., NAD^+^) (E_2_) leaving behind a highly reactive FMN^° -^ that can now transfer the second electron to the more negative electron acceptor (e.g., ferredoxin) (E_1_) (Demmer et al., 2017; Baymann et al., 2018; Buckel and Thauer, 2018a).

**FIGURE 4:**
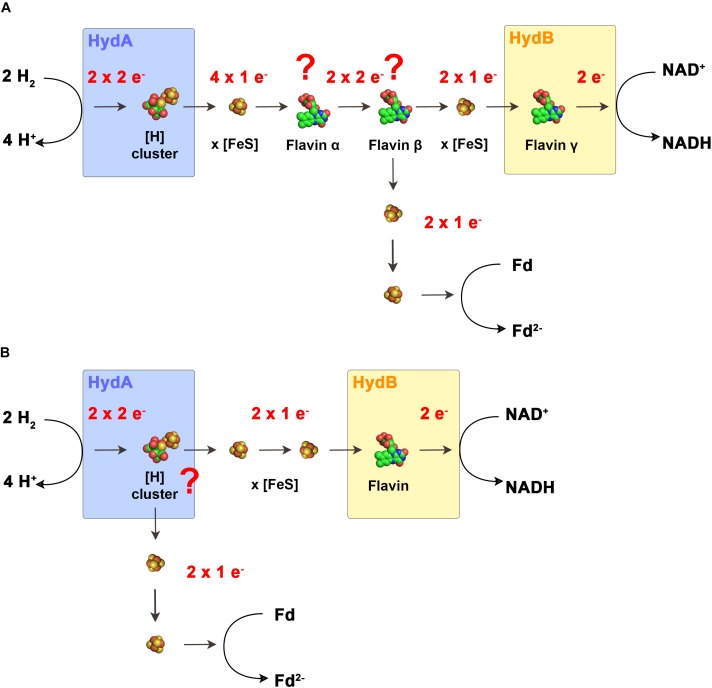
Models for the electron flow in electron bifurcating hydrogenases. In the scenario that electron-bifurcation is facilitated by a flavin **(A)** according to the standard model of FBEB, the enzymes would have to contain two additional flavins that cannot be predicted from the primary structure. Flavin α functions as switch from one electron transferring iron–sulfur clusters to the actual bifurcating flavin β. Here, the two electrons are bifurcated to other iron–sulfur clusters and to the final acceptors. Flavin γ functions as switch from the one electron transferring iron–sulfur clusters to NAD^+^. In the second scenario **(B)**. The H-cluster is the site of electron bifurcation. Here only one flavin is required for the switch from the one electron transferring iron–sulfur clusters to NAD^+^. Fd, ferredoxin.

Based on the current knowledge, FBEB hydrogenases have only one predicted flavin in HydB which is required for the switch from a two-electron carrier (NADH) to a one electron carrier (iron–sulfur cluster), a typical function of “standard” flavins. Therefore, Buckel and Thauer (2013) proposed that FBEB hydrogenases need to have an additional “special” flavin to perform the electron bifurcation reaction. To function in accordance to the standard model of FBEB, the electron flow would look like the following (Figure 4A): H_2_ is oxidized at the H-cluster followed by the electron transfer to the iron–sulfur clusters. H_2_ has a redox potential in between NAD^+^ and ferredoxin, therefore, it must reduce the flavin with a two-electron transfer reaction to reach the flavin redox potential E_3_. However, since iron sulfur clusters can only transfer one electron at a time we therefore speculate that in this model a second flavin (flavin α) would be required for the additional one electron/two electron switch. Then, the “special” flavin (flavin β) can transfer the first electron to the iron sulfur cluster leading to NAD^+^ leaving behind a highly reactive flavin radical that transfers the second electron to the iron–sulfur clusters leading to ferredoxin. The third flavin (flavin γ) is then needed for the second one electron/two electron switch from the iron sulfur clusters to NAD^+^.

Of the characterized FBEB enzymes HdrABC-MvhAGD from *M. thermolithotrophicus*, a complex of a multimeric heterodisulfide reductase and a [NiFe] hydrogenase represents a special case (Wagner et al., 2017). The flavin proposed to be responsible for electron bifurcation is assumed to receive both electrons from H_2_ in two single electron transfer steps in contrast to a two electron transfer from a hydride donor. This is in contrast to the current model of the energetic landscape required for electron bifurcating flavins. However, if electron bifurcation is possible by a flavin connected to three one electron donors/acceptors in the form of iron–sulfur clusters, this could also be the case in FBEB [FeFe] hydrogenases and would render the presence of the hypothetically proposed flavin α redundant.

The aforementioned model assumes the presence of additional flavin binding sites that are not predicted by the amino acid sequence. The flavin content of all isolated FBEB hydrogenases could not be determined or not reported since the flavins are only loosely attached and must be added to the buffers for activity and got immediately lost when left out of the buffers. Assuming that the additional flavins do not exist, a completely new mechanism of FBEB must be present in FBEB hydrogenases. First Nitschke and Russell (2012) raised the possibility that also some metal centers could have crossed redox potentials and could potentially catalyze the same reaction as catalyzed by the flavin cofactor. Peters et al. (2018) took up this exciting possibility and proposed for electron bifurcating hydrogenases that since the FMN cannot be the site for electron bifurcation, the H-cluster could be the possible site for the electron bifurcation reaction. In their proposal, the electrons flow from the H-cluster directly to two different accepting iron–sulfur clusters with different redox potentials. The first string of Fe–S clusters transfers the electrons from the H-cluster to ferredoxin. The other string of iron–sulfur clusters plus the single flavin transfers the electrons from the H-cluster to NAD^+^ (Figure 4B). However, so far no metal center has been shown to accommodate the special properties necessary for electron bifurcation, therefore, this model would lead to a completely new field of enzyme mechanism catalyzed by metal centers.

## The Curious Case of the NAD^+^-Dependent Hydrogenase From *Syntrophomonas wolfei*

**FIGURE 5:**
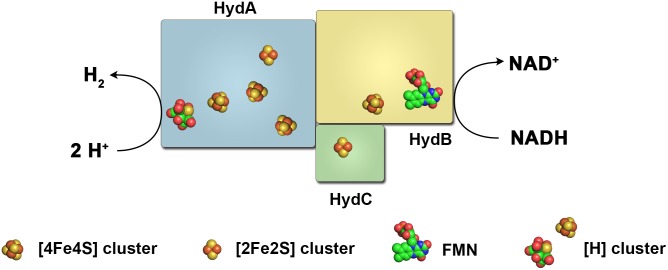
Subunit architecture and cofactor content of the NADH-dependent hydrogenase of *S. wolfei*. This enzyme resembles in the overall subunit architecture electron bifurcating/confurcating hydrogenases that utilize NADH and reduced ferredoxin in an energetically coupled reaction to produce H_2_ (or *vice versa*). The hydrogenase of *S. wolfei* putatively lacks three iron–sulfur clusters in the subunit HydB and utilizes NADH alone for H_2_ production, thus not using FBEB.

Recently, a hydrogenase has been purified from the syntrophic bacterium *S. wolfei* that very much resembles all known FBEB hydrogenases discussed so far, however, does not show FBEB (Losey et al., 2017). Therefore, comparison of this enzyme to the other ones might be helpful to unravel the mechanism of FBEB hydrogenases. The purified recombinant hydrogenase (Hyd1ABC) of *S. wolfei* showed H_2_ production from NADH alone, uncoupled to ferredoxin. The recombinant enzyme had a very high H_2_-dependent: methyl-viologen reducing activity (3,340 U/mg), H_2_-dependent NAD^+^ reducing activity (94.5 U/mg) and catalyzed H_2_ production from NADH with a specific activity of 6.6 U/mg. The enzyme is a trimeric protein complex composed of HydA1 (63 kDa), HydB1 (43 kDa), and HydC1 (17.5 kDa) (Figure 5). It contains five [4Fe–4S], two [2Fe–2S] clusters, and one H-cluster. The flavin content was determined to be 0.7 mol of FMN per mole of enzyme. The Hyd1ABC subunits share close similarity to those of the earlier discussed FBEB hydrogenases. HydA1 is, again, similar to the monomeric *C. pasteurianum* hydrogenase, putatively containing the H-cluster, 3 [4Fe–4S] clusters and one [2Fe–2S] cluster and is the site for H_2_ formation. HydC1 has been predicted to contain one [2Fe–2S] cluster. Nevertheless, the question arose why this enzyme Hyd1ABC of *S. wolfei*, though having the same organization of subunits like FBEB hydrogenases, does not oxidize/reduce ferredoxin. One reason that the authors discuss is the lack of one [2Fe–2S] cluster putatively bound in a N-terminal domain of HydB of FBEB hydrogenases as well as the lack of one [4Fe–4S] cluster putatively bound in the C-terminal domain of HydB of FBEB hydrogenases. In fact, *S. wolfei* HydB1 is much smaller than HydB of other FBEB hydrogenases (43 kDa compared to 63 kDa). Electrons are supposed to be transferred from NADH *via* the FMN to the proximal [4Fe–4S] cluster, and further transferred *via* the [Fe–S] clusters in HydA1 to the H-cluster to reduce protons to H_2_. The *S. wolfei* hydrogenase might be a step in evolution away from FBEB hydrogenases to “standard” hydrogenases or, possibly, the other way around. In any case, solving the structure of this enzyme and comparing it to a bifurcating hydrogenase might unravel factors essential for the energetic coupling within the bifurcating enzymes.

## Formate Dehydrogenase Coupled Hydrogenases

Protons can be used by microorganisms as ubiquitously available electron acceptor to get rid of excess reducing equivalents. A similar function is played by CO_2_. In anoxic environments, CO_2_ or HCO_3_^-^ is ubiquitously available (50–100 mM HCO_3_^-^ in lake sediments, 200-400 mM HCO_3_^-^ in biogas reactors) and can be used as electron acceptor yielding formic acid/formate (Crable et al., 2011; Schink et al., 2017). Interestingly, the redox potential of the CO_2_/formate couple of -432 mV is very close to the H^+^/H_2_ couple rendering both electron acceptors energetically very similar. Therefore, it is not surprising that both H_2_ and formate have been observed as important electron carriers in anoxic environments with often interchangeable functions (Thiele and Zeikus, 1988; Stams and Plugge, 2009; Schink et al., 2017; Montag and Schink, 2018). However, for a long time little was known on how the two pools of high energy redox mediators are connected with each other. In the past years these two compounds gained increasing interest due to their potential as electron donors for biofuel production, as energy carriers for mobile applications or issues like H_2_ storage. This has led to discoveries of novel enzymes but also so far unknown organisms that connect and utilize these two redox mediators.

## Formate Hydrogen Lyase of *E. coli*

Already in 1932 Stephenson and Stickland discovered that whole cells of *E. coli* grown in the presence of formate decompose formate into H_2_ + CO_2_ (Stephenson and Stickland, 1931, 1932). They named the supposed enzyme system formate hydrogen lyase (FHL). When growing under anoxic conditions in the absence of an alternative electron acceptor *E. coli* produces formic acid during mixed acid fermentation from pyruvate catalyzed by pyruvate-formate lyase which is then exported from the cytoplasm by the channels FocA and/or FocB (Clark, 1989; Sawers et al., 2004; Wang et al., 2009; Lu et al., 2011; Trchounian and Trchounian, 2014; Hakobyan et al., 2018). Formic acid (pK_S_ = 3.7) dissociates to formate and leads to an acidification of the environment. A drop in the pH together with the accumulation of formate leads to subsequent import of formate again and the induction of expression of the genes coding for the FHL enzyme. FHL then oxidizes formate to H_2_ + CO_2_ followed by reoxidation of H_2_ by other membrane bound hydrogenases of *E. coli* that transfer the electrons to the quinone pool (Rossmann et al., 1991; Sawers, 1994; Sawers, 2005; Pinske and Sawers, 2016).

**FIGURE 6:**
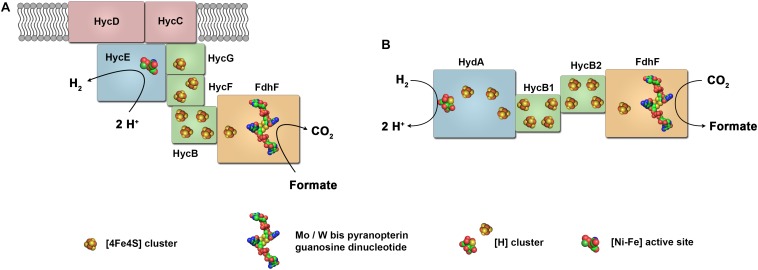
Model of the structure of formate dehydrogenase coupled hydrogenases. FHL of *E. coli* **(A)** has the physiological function of formate oxidation coupled to H_2_ formation. The FHL consist of a [NiFe] hydrogenase coupled to a formate dehydrogenase and membrane integral subunits. In contrast, the HDCR of *A. woodii* **(B)** is composed of a [FeFe] hydrogenase coupled to a formate dehydrogenase and catalyzes reversible CO_2_ reduction with H_2_ as electron donor.

Stephenson and Stickland assumed the enzymes to be a combination of formate dehydrogenase and hydrogenase. Genetic and physiological studies have confirmed this assumption and shown that the FHL of *E. coli* consist of a formate dehydrogenase bound to a membrane integral multimeric hydrogenase located in the cytoplasmic membrane (Zinoni et al., 1986; Böhm et al., 1990; Rossmann et al., 1991; Sauter et al., 1992). However, isolation of the whole FHL complex was achieved only recently by McDowall et al. (2014). The enzyme complex consists of two membrane integral subunits and five soluble subunits. The soluble subunits HycE and HycG represent the large (65 kDa) and small (20 kDa) subunits of the [NiFe]-hydrogenase termed *E. coli* hydrogenase-3 (Hyd-3) (Figure 6A). Formate is oxidized by the subunit FdhF (also called formate dehydrogenase H) (Axley et al., 1990; Gladyshev et al., 1996; Boyington et al., 1997).

The FHL complex shows high similarity to a group of similar membrane bound hydrogenases now called energy-converting hydrogenases (Ech). These enzymes are found in many anaerobic and facultative anaerobic bacteria and H_2_ formation is coupled to different electron donors such as reduced ferredoxin or CO, facilitated by auxiliary subunits (Fox et al., 1996; Künkel et al., 1998; Meuer et al., 1999; Sapra et al., 2000; Soboh et al., 2002). It has been assumed that Ech hydrogenases couple H_2_ formation to the translocation of ions across the membrane. Experimental proof for this concept was established using inverted membrane vesicles of the methanogenic archaeon *Methanosarcina mazei* (Welte et al., 2010a,b). However, whether the FHL complex of *E. coli* is also coupled to energy conservation has been a matter of debate for many years (Bagramyan and Martirosov, 1989; Trchounian et al., 1997, 2000, 2011; Hakobyan et al., 2005; McDowall et al., 2014; Trchounian and Sawers, 2014; Pinske and Sargent, 2016).

FHL couples the two half reactions

(7) $$formate+H^{+}⇌CO_{2}+2e^{-}+2H^{+}\;\;\;\;\;\;\;\;\;\;\;E^{0^{\prime }}=-432\;mV\;$$

and

(8) $$2\;H^{+}+2\;e^{-}⇌H_{2}\;\;\;\;\;\;\;\;\;E^{0^{\prime }}=-414\;mV$$

resulting in the net reaction

(9) $$formate+H^{+}⇌H_{2}+CO_{2}$$

Under standard conditions, the free energy change of Eq. (9) is only -3.5 kJ mol^-1^ (Thauer et al., 1977). Thus, based on the ΔG^0^’ value, the FHL reaction should be fully reversible under physiological conditions, however, the enzyme shows a strong bias toward formate oxidation. Values for *in vitro* turnover frequency (TOF) for FHL activity was reported between 1,200 h^-1^ and 1,920 h^-1^ (McDowall et al., 2014; Pinske and Sargent, 2016) whereas the reverse reaction, formate formation from H_2_ + CO_2_ was only observed with an apparent TOFs of only 202 h^-1^ (measured in a discontinuous one point assay and reported as 3.25 μmol formate produced after 5 h with 370 μg of enzyme) (Pinske and Sargent, 2016). This bias could not be explained by the catalytic properties of FdhF and Hyd-3 that have been analyzed separately and show high activities in both reaction directions (Bassegoda et al., 2014; McDowall et al., 2014). In addition to both active sites a factor determining the bias in one or the other direction could be the thermodynamic landscape of the connecting iron–sulfur clusters. In addition, the membrane attachment of this enzyme complex and the connection to the enzyme activity is still puzzling (Bagramyan and Martirosov, 1989; Trchounian et al., 1997, 2000, 2011; Hakobyan et al., 2005; McDowall et al., 2014; Trchounian and Sawers, 2014; Pinske and Sargent, 2016). From a physiological point of view, the bias could be a remnant of evolution since there is no physiological situation where *E. coli* would use the reverse reaction to produce formate from CO_2_ since this organism is not able to utilize formate further in the metabolism.

## Hydrogen-Dependent CO_2_ Reductases

For many years, FHL was the only enzyme complex known that connects the electron carriers H_2_ and formate but this enzyme shows a strong bias toward formate oxidation. Did evolution also bring up enzymes adapted for the reverse reaction? Efficient catalysts for hydrogenation of CO_2_ are highly sought-after (Appel et al., 2013; Beller and Bornscheuer, 2014; Wang et al., 2015). These could be used for CO_2_ conversion technologies for carbon capture, for CO_2_-based synthesis or for H_2_ storage (Yishai et al., 2016; Preuster et al., 2017b; Bulushev and Ross, 2018). Many homogeneous and heterogenous chemical catalyst have been developed, however, often requiring high temperatures and pressures (Fujita et al., 2013; Wang et al., 2015). In biological systems, of the six pathways known for CO_2_ fixation, only the WLP proceeds *via* direct reduction of CO_2_ to formate catalyzed by formate dehydrogenases (Fuchs, 2011). In 2013, a formate dehydrogenase was isolated from the acetogenic bacterium *A. woodii* that was in complex with a hydrogenase, on first glance resembling the FHL of *E. coli* (Schuchmann and Müller, 2013). However, in contrast to FHL, the enzyme was lacking membrane integral subunits. When isolated, the enzyme contained only four subunits, one formate dehydrogenase, one hydrogenase and two iron–sulfur cluster rich subunits, identified as being encoded by genes found in one gene cluster (Figure 6B). The gene clusters contains in addition a gene coding for a second formate dehydrogenase, a gene for a third iron–sulfur cluster rich subunits and a gene designated as *fdhD* (Poehlein et al., 2012; Schuchmann and Müller, 2013). The formate dehydrogenase FdhF2 as found in the isolated enzyme is a 80.1 kDa protein with 59% identity to FdhH of the *E. coli* FHL aligning over the whole sequence of 713 amino acids. It has one predicted [4Fe–4S] cluster and a bis-PGD cofactor. ICP-MS identified 0.6 mol molybdenum but no tungsten per mol of isolated enzyme preparation agreeing with a mononuclear molybdenum bound to the bis-PGD cofactor thus classifying this enzyme as member of the dimethylsulfoxide reductase family within the Mo/W-bis PGD super-family (Schuchmann and Müller, 2013). At amino acid position 139, a selenocysteine is predicted which is in agreement with the determined selenium in the preparation. In contrast to *E. coli* FHL, the [NiFe] hydrogenase subunits of FHL are substituted by the [FeFe] hydrogenase subunit HydA2 (50.2 kDa). The sequence of this subunit contains the conserved regions typical for H-cluster binding and aligns to amino acid region 201–572 of the monomeric ferredoxin-dependent [FeFe] hydrogenase of *C. pasteurianum* (Figure 3). In contrast to the *C. pasteurianum* enzyme, the N-terminus containing one [2Fe–2S]- and one [4Fe–4S] cluster is missing. In addition to the H-cluster including the adjacent [4Fe–4S] cluster, the enzyme is predicted to contain two additional [4Fe–4S] clusters. HydA2 and FdhF2 are supposed to be connected by the two small electron transferring subunits HycB2 and HycB3 (18.9 and 20.1 kDa, respectively) containing 4 [4Fe–4S] clusters each (Figure 6B).

Enzymatic assays utilizing methyl viologen demonstrated that both FdhF2 as well as HydA2 were highly active in the isolated enzyme. H_2_ oxidation was catalyzed with a TOF of 30,474 s^-1^ (Schuchmann and Müller, 2013). The formate dehydrogenase subunit catalyzed formate oxidation with 1,693 s^-1^ and CO_2_ reduction with 372 s^-1^ also showing a bias for formate oxidation, however, not as severe as reported for FdhH of the FHL that showed no measurable CO_2_ reduction activity with soluble electron carriers. HydA2 has been analyzed by catalytic protein film electrochemistry by adsorbing the whole enzyme complex to an electrode (Ceccaldi et al., 2017). The hydrogenase is biased for proton reduction oxidizing H_2_ 10 times slower than it reduces protons at pH 6 as is typical for [FeFe] hydrogenases. However, this ratio defining the bias decreases with increasing pH.

K_M_ for H_2_ was determined to 0.24 atm [K_i_ (H_2_) = 6.4 atm], being three times lower than reported values for *C. reinhardtii* and *Clostridium acetobutylicum* and thus showing the highest affinity ever reported for a [FeFe] hydrogenase (Fourmond et al., 2013; Ceccaldi et al., 2017). In addition, HydA2 showed the surprising characteristic of being the first completely CO tolerant [FeFe] hydrogenase. Even though the enzyme is strongly inhibited by CO [K_i_(CO) = 0.11 μM], it fully reactivates upon removal of CO which is in contrast to other [FeFe] hydrogenases where CO provokes irreversible damage to the H-cluster (Goldet et al., 2009; Baffert et al., 2011; Foster et al., 2012). This feature could be a result of the metabolism of *A. woodii* where CO is an intermediate of the reductive acetyl-CoA pathway (Schuchmann and Müller, 2014; Bertsch and Müller, 2015b).

Coupling of the formate dehydrogenase/hydrogenase complex of *A. woodii* was analyzed by incubating the isolated enzyme in the presence of formate. This lead to H_2_ production with a TOF of 142,212 h^-1^ (Schuchmann and Müller, 2013). Interestingly, this enzyme is fully reversible also catalyzing hydrogenation of CO_2_ at 30°C and 0.8 bar of H_2_ and 0.2 bar of CO_2_ with a TOF of 101,600 h^-1^ being significantly faster than the currently best known chemical catalysts (Hull et al., 2012; Jeletic et al., 2013; Wang et al., 2015; Eppinger and Huang, 2017). To distinguish the soluble enzyme complex from membrane bound FHL complexes that are weak catalysts for CO_2_ hydrogenation the enzyme was named hydrogen-dependent CO_2_ reductase (HDCR). As mentioned before, CO is a strong inhibitor of the hydrogenase activity (Goldet et al., 2009). Interestingly, the HDCR has an alternative electron entry site and can use reduced ferredoxin as electron donor to allow CO_2_ reduction even in the presence of 1 atm CO. However, this activity is 17 times slower than the hydrogen-dependent activity with a TOF of only 6,095 h^-1^ (Schuchmann and Müller, 2013). Noteworthy, further characterization of the HDCR revealed another interesting property of the enzyme. *In vitro* the HDCR of *A. woodii* reversibly polymerized into ordered filamentous structures of more than 0.1 μM in length (Schuchmann et al., 2016). Divalent cations could be identified to promote the polymerization process and it was observed that the polymerized form of the enzyme was more active. The *in vivo* significance of this observation is unresolved.

As described before, the HDCR gene cluster of *A. woodii* contains a gene, *fdhF1*, for a second formate dehydrogenase subunit with the corresponding electron transferring subunit *hycB1*. The deduced amino acid sequence of FdhF1 is to 80% identical to FdhF2 with the major exception being a cysteine in FdhF1 at position 139 where a selenocysteine is encoded in FdhF2. Metal-dependent formate dehydrogenases have been described with either selenocysteine or cysteine in the active site (Axley et al., 1990; Friedebold and Bowien, 1993; Raaijmakers et al., 2002; de Bok et al., 2003; Laukel et al., 2003). The lower pK_a_ value of selenocysteine compared to cysteine is typically connected to a higher reactivity (Stadtman, 1996; Böck et al., 2005; Stock and Rother, 2009). Exchanging selenocysteine by cysteine in FdhH of *E. coli* let to a decrease in turnover number by over two orders of magnitude. However, the affinity for the substrate was increased in the cysteine variant as reflected by a lower K_M_ value (Axley et al., 1991). There is no data on the different properties of FdhF1 and FdhF2 of *A. woodii*, however, the assumption is that in dependence on the presence of selenium in the environment the two subunits are produced differentially with the selenocysteine containing variant being the more active one. In another acetogenic bacterium, a spirochete from the termite gut, *Treponema primitia*, a gene cluster with similarity to the HDCR gene cluster was identified (Matson et al., 2010). It consists of the genes *fdhF_cys_*, two *hycB* genes, *fdhD* and *hydA*. Separated by 14 genes, a second *fdhF* gene is encoded, containing a putative selenocysteine codon and a SECIS element (*fdhF_sec_)*. Transcript analysis revealed a differential expression of *fdhF_cys_* and *fdhF_sec_* in dependence of selenium availability with up to 40-fold change in transcript levels. Half-maximum decrease in transcript level of *fdhF_cys_* was observed with less than 50 pM sodium selenite, whereas 1.5 nM sodium selenite were required for half-maximum increase in *fdhF_sec_* transcript levels. Interestingly, only transcript levels downstream of the SECIS element were differentially expressed, whereas transcript upstream of the SECIS element did not show differential regulation (Matson et al., 2010).

## Physiology and Diversity of HDCR Complexes

The first HDCR complex has been identified in the acetogenic bacterium *A. woodii*. Acetogenic bacteria utilize the WLP for energy conservation and carbon fixation, the only carbon fixation pathway that utilizes CO_2_ by direct reduction to formate. Therefore, this first reduction step of CO_2_ is essential for the metabolism of these bacteria. The redox potential of CO_2_/formate of -432 mV limits the number of possible electron donors for this reaction. The first formate dehydrogenase characterized in an acetogenic bacterium was isolated from *Moorella thermoacetica* (Yamamoto et al., 1983). This heterotetrameric enzyme was the first enzyme described to contain tungsten and catalyzes CO_2_ reduction coupled to NADPH oxidation. The standard redox potential of NADP^+^/NADPH of -340 mV is too positive for CO_2_ reduction, however, in anaerobes the intracellular ratio of NADP^+^/NADPH is 1/40 resulting in a redox potential of -370 mV (Sauer et al., 2004; Bennett et al., 2009). Insights into an increasing number of sequenced genomes of acetogenic bacteria has revealed that the genes encoding enzymes for the first reaction step of the WLP show a large diversity (Schuchmann and Müller, 2014; Bertsch and Müller, 2015a; Bengelsdorf et al., 2016). Therefore, the knowledge of the enzymes of *M. thermoacetica* could not be transferred to other acetogens. Characterization of *A. woodii* has revealed that it does not use NADPH but H_2_ as electron donor for CO_2_ reduction catalyzed by the HDCR. Energetically, the difference between NADPH and H_2_ is very small under physiological conditions. H_2_ is a stronger reductant under standard conditions (E^0^’ = -414 mV), however, at the minimum H_2_ pressures required by *A. woodii* to perform acetogenesis (250 Pa) the redox potential is only -340 mV (Poehlein et al., 2012). Under these conditions, the equilibrium concentration of formate is 0.1 mM (CO_2_ at 0.2 × 10^5^ Pa, 30°C), in the range of the K_M_ value of next enzyme of the pathway, the formyl-THF synthetase (Poehlein et al., 2012). Coupling CO_2_ reduction directly to H_2_ oxidation also energetically couples it directly to the external H_2_ partial pressure. A H_2_ pressure of 250 Pa is very high compared to the values observed in methanogenic environments of 1 to 10 Pa H_2_ (Conrad et al., 1986; Seitz et al., 1990). Therefore, CO_2_ reduction by HDCR enzymes seems to be not competitive in methanogenic environments. However, the observation of reduced ferredoxin as alternative electron carrier could be a mechanism to overcome this limitation (in addition to the proposed bypass for CO inhibition of the hydrogenase as discussed before). On the other hand, utilizing reduced ferredoxin instead of H_2_ would result, in *A. woodii*, in less ATP conserved (Schuchmann and Müller, 2013). The physiological relevance of the ferredoxin entry site of the HDCR has, however, not been studied yet.

The complete reversibility of the HDCR, as opposed to membrane bound FHL complexes, is a direct reflection of its physiological function. Besides H_2_ + CO_2_, *A. woodii* can grow for example with formate or methanol as sole carbon and energy sources (Schuchmann and Müller, 2016; Kremp et al., 2018). To convert these compounds, part of the substrate must be oxidized to CO_2_ to provide reducing equivalents. In these scenarios, the HDCR must operate in reverse to oxidize formate to H_2_ + CO_2_ since it is the only formate dehydrogenase found in *A. woodii* (Poehlein et al., 2012)_._

**FIGURE 7:**
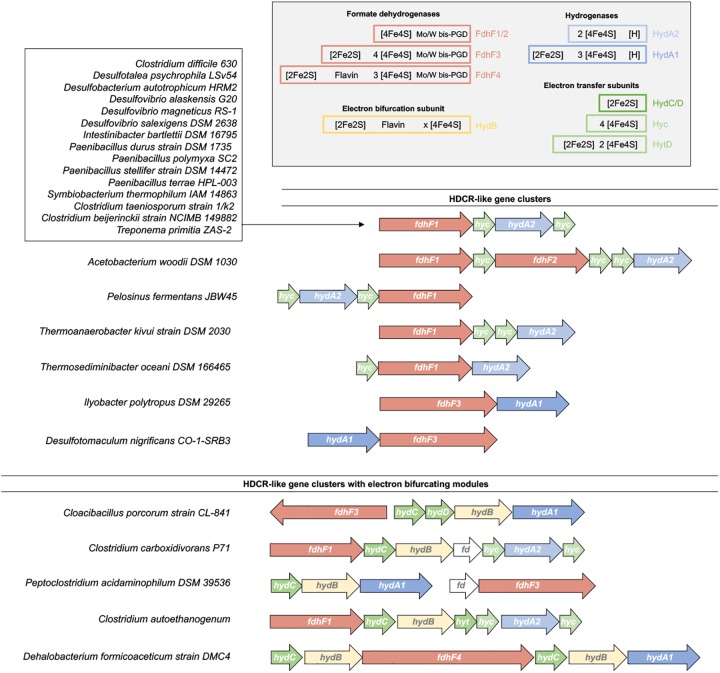
Distribution of HDCR-like gene clusters. Fully sequenced bacterial genomes have been searched for HDCR-like gene clusters (Schwarz et al., 2018). Based on the gene cluster arrangement, the enzymes can be classified in HDCR-like enzyme complex without and with electron bifurcating modules. Predicted cofactors based on the primary sequence of each subunit are shown in the right top corner. For clarity, single genes disrupting the gene cluster in some organisms such as maturation genes (e.g., *fdhD*) or transposase are not shown.

H_2_ and formate are widely used electron carriers in anoxic ecosystems and are product or substrate of many microorganisms. The discovery of the HDCR enzyme as soluble, not energetically coupled enzyme complex that connects these two pools raised the question of the distribution of similar enzymes in other organisms. When searching for gene clusters containing homologs of *fdhF1, hycB1/3* and *hydA2* of *A. woodii* we found 18 organisms encoding gene clusters encoding putative HDCRs (Figure 7), see also Schwarz et al. (2018). Of these putative enzymes only one from the acetogen *Thermoanaerobacter kivui* has been isolated and characterized (Schwarz et al., 2018). Of the 18 organisms, three are acetogenic bacteria that can grow with H_2_ + CO_2_ as substrates [*Clostridium difficile* (Köpke et al., 2013), *T. primitia* (Graber and Breznak, 2004), *T. kivui* (Leigh et al., 1981)] and 5 belong to the sulfate reducers [*Desulfotalea psychrophila* (Knoblauch et al., 1999), *Desulfobacterium autotrophicum* (Brysch et al., 1987), *Desulfovibrio alaskensis* (Feio et al., 2004), *Desulfovibrio magneticus* (Sakaguchi et al., 2002), *Desulfovibrio salexigens* (Postgate and Campbell, 1966)]. In these organisms the HDCRs could play a role in the WLP as in *A. woodii* to reduce CO_2_ to formate either for carbon fixation alone (sulfate reducers) or for carbon fixation and energy conservation (acetogens). Many organisms from the genus *Paenibacillus* have putative HDCR gene clusters. Paenibacilli are found in many environments from polar to tropic regions, often found in soil where they have been reported to be associated with plant roots promoting plant growth, some species are pathogenic to honeybees or invertebrates and some are opportunistic pathogenic to humans (Grady et al., 2016). Species such as *Paenibacillus polymyxa* are facultative anaerobes and can ferment glucose under anoxic conditions by mixed acid fermentation to a mixture of products such as acetate, ethanol, lactate, formate, H_2_ and CO_2_ (Marwoto et al., 2004). Cell free extracts of *P. polymyxa* have been shown to contain hydrogenase activity and catalyze H_2_ evolution from formate (Grau and Wilson, 1962). We conclude, that in Paenibacilli the HDCR could take over the function of the FHL complex as used by *E. coli* to detoxify formate produced during fermentation by oxidizing it to H_2_ + CO_2_.

We identified two gene clusters that show notable differences to the standard HDCR gene cluster. In this case, the hydrogenase subunit resembles the hydrogenase subunit of electron bifurcating hydrogenases by containing two additional iron–sulfur clusters. The formate dehydrogenase subunit also contains three putative additional iron–sulfur clusters whereas genes for the Hyc proteins are missing (Figure 7). The additional iron–sulfur clusters in the Hyd and Fdh subunit could take over the function of electron transfer otherwise catalyzed by the Hyc proteins. In contrast, in five other organisms we identified putative HDCR gene cluster that are more complex than the “standard” HDCR and that have, in the case of *Clostridium autoethanogenum*, been shown to combine features of HDCR-like enzyme complexes and electron bifurcating hydrogenases (Wang et al., 2013a).

## Raising the Complexity: Formate Dehydrogenase Coupled Electron Bifurcating Hydrogenases

**FIGURE 8:**
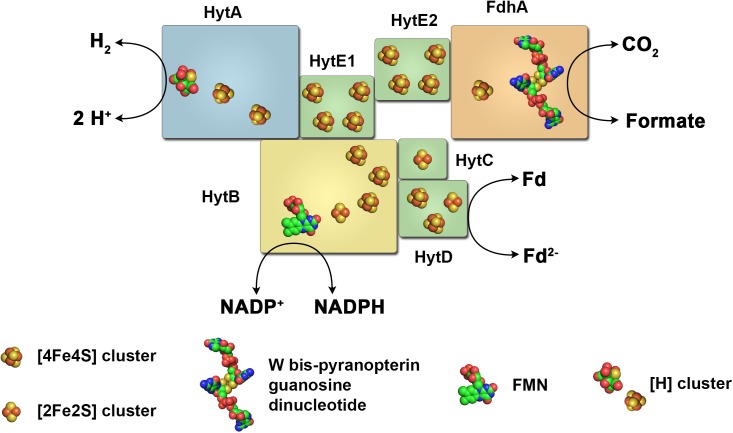
The electron-bifurcating formate dehydrogenase/hydrogenase complex of *C. autoethanogenum*. The enzyme complex of the acetogenic bacterium *C. autoethanogenum* consists of a [FeFe] hydrogenase, a formate dehydrogenase and subunits typical for electron bifurcating hydrogenases. It catalyzes electron transfer from H_2_ or formate to NADP^+^ and ferredoxin (energetically coupled) and *vice versa*, or from H_2_ to CO_2_ forming formate and *vice versa*. Fd, ferredoxin.

In the acetogenic bacterium *C. autoethanogenum* a very complex enzyme has been isolated that indeed combines the features of the HDCR enzyme complex and hydrogenases utilizing FBEB (Wang et al., 2013a). This complex is encoded by seven genes found in one cluster on the chromosome. It putatively contains 19 iron–sulfur clusters that could connect the active site of a [FeFe] hydrogenase and a selenocysteine containing bis-PGD containing formate dehydrogenase (Figure 8). In addition, a flavin is predicted to be bound to the complex. Chemical analysis of the isolated complex confirmed the presence of FMN, selenium, tungsten and 60 mol iron of 76 predicted mol iron per complex. Molybdenum and FAD were not detected. The enzyme complex showed very interesting catalytic properties. When incubated with H_2_ + CO_2_ it catalyzed formate formation with 41 U mg^-1^ and the reverse reaction, H_2_ formation from formate with 40 U mg^-1^ thus showing the typical reversible reaction of HDCR enzymes. However, when incubated with H_2_, NADP^+^, and oxidized ferredoxin the enzyme oxidized H_2_ and reduced NADP^+^ and ferredoxin simultaneously (32 U mg^-1^). NADP^+^, NAD^+^ or ferredoxin alone in the presence of H_2_ are not reduced. H_2_ could also be formed only in the presence of NADPH and reduced ferredoxin together (27 U mg^-1^). In addition, NADP^+^ and oxidized ferredoxin could also be reduced with formate, again only in the presence of both electron acceptors (15 U mg^-1^). The same was true for the reverse reaction, CO_2_ reduction with NADPH and reduced ferredoxin (7 U mg^-1^). It has been further demonstrated that the electron-bifurcating reactions with two electron acceptors are strictly energetically coupled (Wang et al., 2013a). Taking these results together, the enzyme represents a combination of formate dehydrogenase coupled hydrogenase as in the case of the HDCR and subunits facilitating FBEB. Interestingly, electron bifurcation to NADP^+^ and oxidized ferredoxin is catalyzed with two alternative electron donors, H_2_ or formate. Puzzling as well is the observation that CO inhibits not only the hydrogenase activity but also reduction of NADP^+^ and ferredoxin with formate, even though CO is not known to inhibit formate dehydrogenases. The physiological function is complex as well. The enzyme is highly produced in cells grown with syngas as substrate (42% CO, 36% N_2_, 20% CO_2_, and 2% H_2_) even though already CO concentrations of around 0.1% inhibit most activities of the enzyme complex to 50%. Wang et al. (2013a) propose that during balanced growth on CO the steady state concentration within the cell is much lower than external CO concentrations thus not inhibiting the enzyme completely. The authors propose the enzyme utilizes NADPH and reduced ferredoxin for CO_2_ reduction when cells grown on CO and the hydrogenase module has the function to protect the cells from over-reduction when NADP^+^ and ferredoxin get too reduced during growth of *C. autoethanogenum* on CO. For a detailed discussion of this function we refer the reader to the original work of Wang et al. (2013a) since it is beyond the scope of this review.

In four other organisms, we identified gene clusters that encode for putative enzyme complexes resembling the electron-bifurcating formate dehydrogenase/hydrogenase complex of *C. autoethanogenum*. These include the acetogen *Clostridium carboxidivorans* that can, as *C. autoethanogenum* utilize CO as carbon and energy source (Liou et al., 2005) and the amino acid fermenting bacteria *Cloacibacillus porcorum* (Looft et al., 2013) and *Peptoclostridium acidaminophilum* (formerly *Eubacterium acidaminophilum*) (Zindel et al., 1988) (Figure 7). *P. acidaminophilum* uses formate as electron donor to reduce glycine, sarcosine, or betaine to acetyl phosphate (Hormann and Andreesen, 1989; Andreesen, 1994). In addition, it is able to grow in syntrophic culture on, e.g., alanine, valine, leucine or malate if a hydrogen- or formate-consuming bacterium is also present (Zindel et al., 1988). The genome encodes for two putative formate dehydrogenases of which one has been isolated and characterized (Graentzdoerffer et al., 2003). The purified enzyme contained the subunits FdhF (selenocysteine and tungsten bis-PGD cofactor containing formate dehydrogenase), two iron–sulfur cluster containing subunits and the flavin- and NAD(P) binding subunit HydB. The corresponding gene cluster encodes for a [FeFe] hydrogenase, however, hydrogenase activity was lost during purification and a hydrogenase subunit or hydrogenase activity could not be detected in the isolated enzyme. On the other hand, formate dehydrogenase activity was present and showed reversible catalysis of formate oxidation/CO_2_ reduction (43 U mg^-1^/12 U mg^-1^, methyl viologen as electron donor/acceptor).

## Biotechnological H_2_ Production and Storage

H_2_ has been considered for a long time to replace fossil fuels in the future to tackle the climate problem by decreasing emission of CO_2_ (Ball and Wietschel, 2010). H_2_ is a very simple molecule that can be produced by various methods including splitting of water that, if the energy is provided by renewable energy sources like solar or wind power does not lead to CO_2_ emission. Consumption of H_2_ for energy generation either by thermal combustion or H_2_ fuel cells at the site of use does not release CO_2_ as well. On the other hand, significant challenges are connected with a switch to the H_2_ economy. Currently, 95% of world-wide H_2_ is produced from fossil fuels by coal gasification or by steam reforming of natural gas followed by further processing to increase the H_2_ yield (water-gas shift reaction) (Brandon and Kurban, 2017). These processes emit large quantities of CO_2_ and are not sustainable. Another major drawback of H_2_ is its very low density. On a gravimetric basis, the energy content of 33.3 kWh/kg H_2_ is three times higher than that of gasoline. On a volumetric basis this situation is dramatically reversed to 3 Wh/L for gaseous H_2_ versus 8,600 Wh/L to 9800 Wh/L for gasoline or diesel, respectively (Preuster et al., 2017a). Therefore, methods need to be developed for on-board storage of H_2_ in mobile applications such as cars and large-scale storage of H_2,_ e.g., for storing energy produced by wind or solar power at off peak times. Another challenge of H_2_ concerns safety issues with respect to the high volatility and the formation of highly explosive gas mixtures if getting in contact with air.

A key technology to sustainably produce H_2_ without carbon emission is electrolysis of water driven by electricity generated from renewable sources (Ursua et al., 2012). Electrocatalytic splitting of water releases only H_2_ and O_2_ thus generating H_2_ free of contaminations such as CO which is important for H_2_ fuel cell applications. This process has been used already in large scale, however, the low price of fossil fuels has rendered it uneconomic. In the near future, process efficiencies of 85–95% are expected with the current price of H_2_ produced by electrolysis being approximately at 3 € per kg (Tremel et al., 2015).

Biological catalysis provides another alternative method for H_2_ production. These processes can currently not be considered as the major technology to provide H_2_ for the H_2_ economy but could provide small but significant contributions to exploit other H_2_ sources as well. H_2_ production by biological systems can be classified into four general mechanisms, namely direct and indirect biophotolysis, photofermentation, and dark fermentation which have been extensively reviewed elsewhere (Manish and Banerjee, 2008; van Niel, 2016; Kumar et al., 2018). We want to focus here on dark fermentation which has shown the highest H_2_ evolution rates so far (Rittmann and Herwig, 2012), biological alternatives to store H_2_ and the new opportunities provided by the recent discoveries of novel types of hydrogenases.

Dark fermentation can be catalyzed by single organisms or complex consortia to extract energy from multiple substrates such as waste biomass. This process has been used successfully for many years in biogas plants that convert waste biomass to methane. Within this process, H_2_ is produced by fermenting organisms but immediately consumed by methanogens. Selective inhibition of methanogens can stop H_2_ consumption leading to H_2_ accumulation (Schink, 1997; Catal et al., 2015). Alternatives are single organisms capable of producing large quantities of H_2_ either naturally or by metabolic engineering. In nature, most H_2_ is produced from the fermentation of carbohydrates (Thauer et al., 2008). Complete oxidation of 1 mole of hexoses such as glucose would yield a maximum of 12 moles of H_2_

(10) $$C_{6}H_{12}O_{6}+12\;H_{2}O⇌12\;H_{2}+12\;HCO_{3}^{-}$$

This reaction is endergonic [ΔG^0^’ = 13.6 kJ mol^-1^ (Flamholz et al., 2012)] and thus not feasible *in vivo*. The maximum H_2_ that can be produced from glucose by microorganisms is limited to 4 mol H_2_ per mol of glucose known as the Thauer limit (Thauer et al., 1977). In practice, most H_2_ yields of microorganisms are far below this theoretical limit due to thermodynamic reasons. Oxidation of glucose to 2 pyruvate by the Embden–Meyerhof–Parnas pathway transfers all electrons to NAD^+^ producing in total 2 NADH. In contrast, oxidative decarboxylation of 2 pyruvate to acetyl-CoA and CO_2_ can be used to reduce 2 ferredoxins as electron carrier. H_2_ production from ferredoxin is exergonic whereas H_2_ production from NADH is endergonic as described above. Thus, without a H_2_ consuming partner organism, H_2_ can only be produced from reduced ferredoxin resulting in a maximum of 2 H_2_ produced per mol of glucose. The solution to this problem is provided by the new class of electron-bifurcating hydrogenases that can produce H_2_ from NADH as well, driven by the coupled production of H_2_ from reduced ferredoxin (Buckel and Thauer, 2013). Organisms that harbor such enzymes such as *T. maritima* (Selig et al., 1997; Frock et al., 2012) or *R. albus* (Zheng et al., 2014) are able to reach the Thauer limit by fermenting glucose according to

(11) $$Glucose+2\;H_{2}O⇌4\;H_{2}+2\;Acetate^{-}+2\;CO_{2}$$

with a ΔG^0^’ value of -250 kJ mol^-1^ at 90°C (growth temperature of *T. maritima*) and -215 kJ mol^-1^ at 25°C. An alternative strategy to reach these high H_2_ yields has been observed in the hyperthermophilic archaeon *Pyrococcus furiosus* growing at 100°C. In this organism the NAD^+^-dependent glyceraldehyde-3-phosphate dehydrogenase is replaced by a ferredoxin-dependent enzyme at the expense of this enzyme not being couple to ATP formation (Mukund and Adams, 1995). Thus, glucose oxidation results only in generation of reduced ferredoxin allowing full reoxidation by formation of H_2_.

Because of the good understanding of the metabolism and the well-established genetic tools, *E. coli* is the prime candidate for biotechnological applications. However, *E. coli* does not harbor an FBEB hydrogenase and is limited to producing 2 mol H_2_ per mol glucose. Recently, it has been demonstrated for the first time that heterologous expression of an FBEB hydrogenase in *E. coli* is possible (Kelly et al., 2015). Since *E. coli* lacks pyruvate:ferredoxin oxidoreductase (PFO), this enzyme from *T. maritima* as well as ferredoxin from *T. maritima* had to be produced additionally in *E. coli* to achieve H_2_ production. The amounts and rates of H_2_ production were very low therefore this study can be seen as mere proof of principle that such a complex hydrogenase can be produced in *E. coli*, however, future studies need to prove that this approach indeed can significantly improve the H_2_ yields of *E. coli*.

The recently discovered formate dehydrogenase coupled hydrogenases could also contribute to the H_2_ economy in the part of H_2_ storage and transportation. To overcome the very low volumetric energy density of gaseous H_2_ many technologies are tested to store H_2_ in a more compact form (Schlapbach and Zuttel, 2001; Preuster et al., 2017a,b). Physical options are compression and storage in high pressure tanks above 200 bar or storage of H_2_ in its liquid form present at -253°C, both technologies requiring high energy input reducing the efficiency of H_2_ as energy carrier. A chemical alternative is reacting H_2_ with other compounds to produce liquid organic H_2_ carriers (LOHCs). For example, hydrogenation of CO_2_ leads to formic acid that contains 4.4 wt % of H_2_. This is close to the 2017 H_2_ storage target of 5.5 wt % in gravimetric energy density set by the United States Department of Energy at temperatures from -40 to 60°C at a maximum pressure of 100 atm (Enthaler et al., 2010; Laurenczy, 2011; Kawanami et al., 2017). The volumetric capacity is 53 g H_2_/L formic acid thus one liter formic acid can store roughly 600 liter of gaseous H_2_. Formic acid is non-toxic and non-explosive, however, its corrosive nature requires special consideration for tanks and equipment. Formic acid can be decomposed to H_2_ + CO_2_ before H_2_ is then used in a H_2_ fuel cell. Chemical catalysts have been developed that catalyze this dehydrogenation with high activity and stability at ambient temperatures below 100°C (Boddien et al., 2008, 2009). However, the initial hydrogenation of CO_2_ still represents a challenge. Many homogenous and heterogenous chemical catalyst have been developed but are depending on either high temperatures and pressures, expensive bases or special media for high efficiencies (Hull et al., 2012; Jeletic et al., 2013; Wang et al., 2015; Eppinger and Huang, 2017). Highest rates for CO_2_ hydrogenation (TOF 3,400 h^-1^) with chemical catalyst at ambient conditions have been achieved with cobalt based catalysts in the presence of a special and expensive base (Verkade’s base) (Jeletic et al., 2013). The newly discovered HDCR complexes could provide a biotechnological alternative or could serve as model to design more efficient catalysts. HDCR of *A. woodii* catalyzes CO_2_ hydrogenation with a TOF of 101,600 h^-1^ at 30°C and 1 bar of pressure (Schuchmann and Müller, 2013). In contrast to other CO_2_ reductases the enzyme directly utilizes H_2_ thus not requiring soluble electron carriers. In addition, with even higher TOFs it catalyzes the reverse reaction as well, closing the cycle for a H_2_ storage process. By coupling the HDCR with a ferredoxin dependent CO dehydrogenase, conversion of syngas (H_2_, CO, CO_2_) or CO alone to formate was achieved (Schuchmann and Müller, 2013). This is advantageous since CO is very toxic to H_2_ fuel cells and can be removed by intermittent conversion of the gases to formate. Major drawbacks are the high oxygen sensitivity and inherent stability issues typical for biocatalysts such as narrow pH and temperature range. To overcome the requirement of enzyme isolation, a whole cell system has been established using *A. woodii* as catalyst for reversible H_2_ storage (Schuchmann and Müller, 2013). Genetic manipulations were not required since competing pathways for product formation were inhibited by addition of a sodium ionophore that makes the cytoplasmic membrane permeable for sodium ions thus inhibiting energy conservation and ATP synthesis. Using this system, formate could be specifically produced from H_2_ + CO_2_ reaching final concentrations (up to ∼0.25 M formate) that where only limited by the thermodynamics of the reaction. Formate was produced with a rate of 120 mmol formate h^-1^ g^-1^. Formate decomposition was catalyzed by whole cells with activities up to 71 mmol H_2_ h^-1^ g^-1^ (Kottenhahn et al., 2018). Yields of 1 mol H_2_ per mol of formate were demonstrated. Recently, the first HDCR from a thermophile, namely the acetogen *T. kivui*, was isolated (Schwarz et al., 2018). This enzyme showed surprising activities with TOFs for CO_2_ hydrogenation of 9,556,000 h^-1^ at 60°C and still 1,856,000 h^-1^ at 30°C. Formate dehydrogenation was catalyzed with a TOF of 9,892,000 h^-1^. This enzyme contained in contrast to the HDCR of *A. woodii* tungsten instead of molybdenum and no selenocysteine in the formate dehydrogenase subunit. Future insights into the structure and biochemistry of this enzyme will hopefully unravel the factors determining this tremendous CO_2_ reductase activities not observed for other apparently very similar formate dehydrogenases.

One alternative for HDCR catalysis of CO_2_ hydrogenation is the FHL enzyme complex as found in *E. coli*. For many years thought to work efficiently only in direction of formate oxidation, recent work has demonstrated the reversibility. Even though CO_2_ reductase activity of the isolated enzyme are orders of magnitude slower compared to HDCR enzymes, whole cell catalysis under high pressure showed promising results (Roger et al., 2018). Utilizing a genetically modified strain that is deficient in competing formate dehydrogenases, hydrogenases and pyruvate formate lyase that are otherwise present in *E. coli* and using 10 bar of H_2_ + CO_2_ (H_2_:CO_2_ ratio 1:1) at 37°C approximately 500 mM formate could be produced over a time course of 23 h. Yields reached 100% (produced formate per consumed CO_2_) and initial rates of 36 mmol formate produced h^-1^ g^-1^ were demonstrated. Even though the FHL enzyme has less advantageous catalytic properties in the isolated form and has the disadvantage of being a membrane integral enzyme complex, the catalytic rates observed with whole cells of *E. coli* in the high-pressure system are in the same order of magnitude as whole cell catalysis with *A. woodii*, however, the latter being performed at ambient pressures.

**FIGURE 9:**
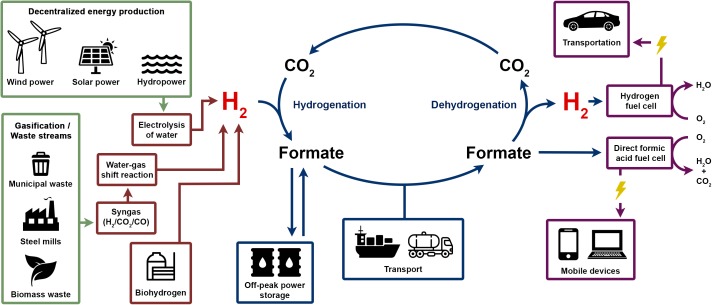
Formate storage and the H_2_ economy and envisioned H_2_ economy could use sustainably produced electricity, biohydrogen or gasified waste as sources for H_2_ gas. Hydrogenation of CO_2_ leads to formic acid/formate which can be stored and transported easier, safer and in a more compact form compared to gaseous H_2_. Formic acid can serve as fuel directly in a direct formic acid fuel cell or formate is dehydrogenated to release H_2_ again to be used in a H_2_ fuel cell in, e.g., fuel cell powered cars.

How could formic acid be integrated in the H_2_ economy? As shown in Figure 9, energy production from renewable sources such as solar or wind power will be more decentralized. Electricity produced could be converted electrochemically by water splitting into H_2_. Alternative H_2_ sources can be biohydrogen produced for example from waste biomass and multiple sources of synthesis gas such as industrial off-gas or gasification of municipal waste or biomass. H_2_ production from syngas are well established technologies utilized already as major pathway for H_2_ production. In the next step, H_2_ could be bound to CO_2_ at decentralized facilities producing formic acid that could then be stored in bulk amounts for energy storage or distributed to the final customer. Formic acid could be used directly for energy generation in direct formic acid fuel cells (DFAFCs) that are potential power sources for portable devices (Aslama et al., 2012). DFAFCs are less well developed compared to H_2_ fuel cells. Therefore, for utilization in H_2_ fuel cells, H_2_ must be generated first by dehydrogenation of formic acid again at the site of use. Combustion of formic acid directly or H_2_ after formic acid dehydrogenation results in release of water and CO_2_ only. CO_2_ can be used for the next H_2_ storage cycle; therefore, no net CO_2_ is generated.

## Conclusion

Enzymes utilizing H_2_ are known for almost a century and since the first crystal structure of a hydrogenase in 1995 (Volbeda et al., 1995) a very large number of studies revealed many facets of the biochemistry, reaction mechanism and evolution of hydrogenases. Most knowledge has been gained about the core subunit of hydrogenases that harbors the active site for H_2_ oxidation/H^+^ reduction, however, in recent years more and more hydrogenases have been discovered that are part of large and very complex multimeric enzymes connecting H_2_ oxidation to multiple functions. One of the major breakthroughs in (anaerobic) microbiology within the last decade was the discovery of the novel enzyme mechanisms of FBEB. This discovery solved many thermodynamic enigmas within the physiology of anaerobic microorganisms. Concerning H_2_ production, unraveling of hydrogenases utilizing FBEB finally solved the long-standing questions how H_2_ production from NADH is possible and, the other way around, how H_2_ can be used to reduce ferredoxin, both highly endergonic reactions without energetic coupling to other redox reactions. These enzymes follow a modular architecture with the large subunit of [FeFe] hydrogenases HydA being coupled to other functional subunits. The same is true for hydrogenases coupled to formate dehydrogenases or the combination of hydrogenase, formate dehydrogenase and electron bifurcation. All these enzymes contain a large number of iron–sulfur clusters that are supposed to accomplish the electrical connection of the different active sites or the active sites and multiple electron acceptors. The biochemical characterization of all these enzymes needs now to be followed by detail insights into the structure and reaction mechanisms. FBEB hydrogenases constitute a fascinating puzzle on how the splitting and energetic coupling of the two electrons is achieved since the flavin to take over this function in other characterized FBEB enzymes is apparently missing. The idea that indeed a metal center can take over this function is a compelling thought. On the other hand, a detailed understanding of H_2_-dependent CO_2_ reductases would not only be important from a biochemical point of view but could also help to provide better catalysts for economic problems such as H_2_ storage. After more than two decades focusing on the major subunit of hydrogenases the next decade might reveal the fascinating complexity of the auxiliary subunits of this still highly important class of enzymes.

## Author Contributions

All authors listed have made a substantial, direct and intellectual contribution to the work, and approved it for publication.

## Conflict of Interest Statement

The authors declare that the research was conducted in the absence of any commercial or financial relationships that could be construed as a potential conflict of interest.
